# Ionic Adsorption and Desorption of CNT Nanoropes

**DOI:** 10.3390/nano6100177

**Published:** 2016-09-28

**Authors:** Jun-Jun Shang, Qing-Sheng Yang, Xiao-Hui Yan, Xiao-Qiao He, Kim-Meow Liew

**Affiliations:** 1 Department of Engineering Mechanics, Beijing University of Technology, Beijing 100124, China; shangjunjun@emails.bjut.edu.cn (J.-J.S.); yanxiaohuiyxh@emails.bjut.edu.cn (X.-H.Y.); 2 Department of Civil and Architectural Engineering, City University of Hong Kong, Tat Chee Avenue, Kowloon, Hong Kong 999077, China; kmliew@cityu.edu.hk

**Keywords:** nanorope, adsorption and desorption of ions, carbon nanotubes

## Abstract

A nanorope is comprised of several carbon nanotubes (CNTs) with different chiralities. A molecular dynamic model is built to investigate the ionic adsorption and desorption of the CNT nanoropes. The charge distribution on the nanorope is obtained by using a modified gradient method based on classical electrostatic theory. The electrostatic interactions among charged carbon atoms are calculated by using the Coulomb law. It was found here that the charged nanorope can adsorb heavy metal ions, and the adsorption and desorption can be realized by controlling the strength of applied electric field. The distance between the ions and the nanorope as well as the amount of ions have an effect on the adsorption capacity of the nanorope. The desorption process takes less time than that of adsorption. The study indicates that the CNT nanorope can be used as a core element of devices for sewage treatment.

## 1. Introduction

With the rapid development of industry, heavy metal pollution and its control have become the major environmental problems [1,2]. Metal plating equipment, petroleum and chemical industry, battery industry, pesticide residues, and so on are producing more and more heavy metal which directly or indirectly emit to the environment [3], especially in the developing world. Heavy metal is different from organic pollutants [4]. When in water, it is not biodegradable and easy to accumulate in organisms. As we all know, Cu^2+^, Hg^2+^, Cd^2+^, and Pb^2+^ are common toxic pollutants. Water, soil [5], and biology are difficult to detoxify once polluted by the heavy metal ions. It has been proved that many heavy metal ions are poisonous and carcinogenic substances and cause serious harm to ecological environments and humans, endangering life and health. Considering these serious public health implications, how to remove heavy metal ions from water has become a major challenge confronting humanity. Moreover, the most detrimental impurities in the feed water are high molar mass substances, except metal ions and organic pollutants. When the content of ions like As^III^, As^V^, F^−^, NO_3_^−^, etc. exceed an acceptable level, these ions are released into the environment, which causes serious environmental and health problems.

One way to remove ions in industrial wastewater is adsorption [6,7], which is simple and inexpensive. Repeated utilization is an important consideration for evaluating the stand or fall of adsorption material. It is required that the materials should not only have good adsorption ability, but also have good desorption ability [8,9]. The structure and properties of carbon nanotubes (CNTs) may make it a good adsorption material [10,11]. CNTs, as a new type of nanomaterials, not only have excellent mechanical, thermal, and electrical properties, but also possess good chemical and thermal stability and a large specific surface area. They have important application value in adsorbing heavy metal ions effectively and in the extraction of minerals, especially rare earth elements [12]. Ajayan et al. [13] found that CNTs can be filled with lead without inducing any damage. When applying voltage to one end of the tubes, extra charges can be attached to tubes and then charge redistribution takes place [14]. The charge properties of CNTs can vary with the amount of charge [15]. The corresponding carbon quantum wires, CNT field emission devices, nanotubes tweezers, and carbon nanotube actuators [14,15] have been developed successively. A series of results show that charged CNTs have enormous development potential [16,17,18]. It has been shown that, due to the additional charges in CNTs, the ionic velocity in NaCl solutions markedly changes [18]. Alijani et al. [19] came up with a new application for magnetic CNTs, namely the removal of lead ions, and the results suggest that this sorbent is suitable for the removal and solidification of metal ions from polluted environmental samples. Doubt studied the changing structure and dynamic behavior of metal salt, Ru, Bi, Ag, Pt, Pd, and biomolecules when they are encapsulated in CNTs [20]. Guo et al. [21] investigated a CNT system decorated with transition metals such as Ni, Cu, Au, and Pt. Valentini et al. [22] concluded that water molecules in CNTs can be influenced by an electric field. Water molecules are very stable and have a high efficient adsorption in electric fields. When the electric field is removed, CNTs can recover their initial state.

Although the adsorption of single CNTs has been widely investigated, their size makes them difficult for industrial applications. CNT assemblies including different CNT-based nanostructures are expected to deliver the superb properties of CNTs from nano-scale to macro-scale. It is important to investigate the adsorption ability of CNT-based nanostructures. Akin to the common geometry of synthetic fiber ropes [23], a model of a CNT nanorope was built by twisting a CNT bundle. In this paper, the ionic adsorption and desorption of CNT nanoropes were realized by changing the strength of the applied electric field. Firstly, molecular dynamic models of CNT nanoropes were built. Then, the charge distribution on the nanorope was obtained by using a gradient method based on classical electrostatic theory. Finally, the adsorption and desorption of the metal ions and negative ions of the charged nanoropes were investigated.

## 2. Molecular Dynamics Model of Nanoropes

**Figure 1:**
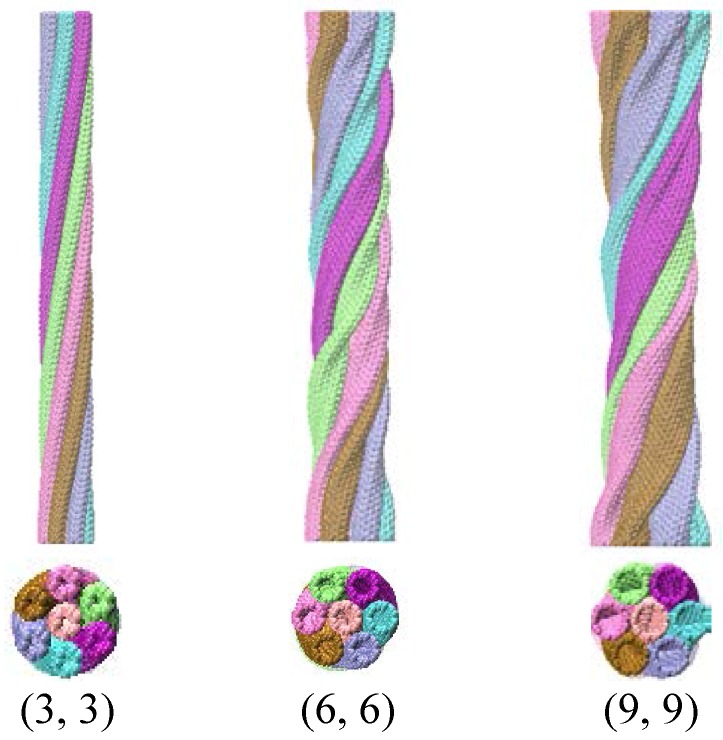
Nanoropes including carbon nanotubes (CNTs) with chiralities (3, 3) (**left**), (6, 6) (**middle**), and (9, 9) (**right**).

A CNT bundle including seven CNTs was generated in the quantum mechanics software Materials Studio. The initial length in the free state was 20.168 nm. Then, the bundle was twisted around a core axis to form a nanorope by using LAMMPS (Large-scale Atomic/Molecular Massively Parallel Simulator) code [24]. In this paper, the Adaptive Intermolecular Reactive Empirical Bond Order (AIREBO) potential function [25,26,27] is used to describe the interaction between carbon atoms. The potential consists of three terms.

(1) $$E=\frac{1}{2}\sum_{i}\sum_{j\neq i}\left[E_{ij}^{\text{REBO}}+E_{ij}^{\text{LJ}}+\sum_{k\neq i,j}\sum_{l\neq i,j,k}E_{kijl}^{\text{TOR}}\right],$$

where $E_{ij}^{\text{REBO}}$ represents the reactive empirical bond-order potential developed by Brenner et al. [26], $E_{ij}^{\text{LJ}}$ represents the longer-ranged interactions in a form similar to the standard Lennard–Jones potential, and $E_{ij}^{\text{TOR}}$ stands for the torsional interaction. The simulation process made use of the NVT ensemble (the system is isolated from changes in moles, volume, and temperature). In order to avoid the effects of thermal activation, the Nose/Hoover thermostat kept the temperature of the system at 1 × 10^−3^ K, and the velocity obeyed the Boltzmann distribution. The time step was set to be 1 fs, and the trajectory information was output every 500 steps. Three-dimensional non-periodic boundary conditions and rotation load were applied. Under the rotation, the CNTs were self-assembled into a nanorope under the action of Van der Waals force. After the full energy relaxation (1 ns) of the system, the model of nanorope was obtained. Figure 1 shows three nanoropes including CNTs with chiralities (3, 3), (6, 6), and (9, 9).

The charge distribution was calculated by using a modified gradient method [28] based on classical electrostatic theory [29,30]. It is assumed that the nanorope is subjected to an ideal electric field, and the charges induced by the electric field distribute on the surface of CNTs. For a nanorope with n atoms, the electric potential at an arbitrary atomic position can be expressed by

(2) $$V\left(r_{i}\right)=\sum_{j=1}^{n}q_{j}/\left(4\pi \varepsilon_{0}\left|r_{i}-r_{j}\right|\right),$$

where $q_{j}$ (*j* = 1, 2, 3 …, n) denotes the charge on each carbon atom, $r_{i}$ and $r_{j}$ represent the location of carbon atoms *i* and *j*, respectively, and ε_0_ is the permittivity of vacuum. *n* equations can be written in a matrix form as following:

(3) $$\left[A\right]\left\{q\right\}=\left[V_{x}\right],$$

where {*q*} and [*V_x_*] stand for charge vector and electric potential vector. [*A*] is a diagonal matrix of *n* × *n* dimension, and its elements can be written as

(4) $$\begin{array}{cc} a_{ij} & =1/\left(4\pi \varepsilon_{0}\left|r_{i}-r_{j}\right|\right)\\ & =1/\left(4\pi \varepsilon_{0}\sqrt{{\left(x_{i}-x_{j}\right)}^{2}+{\left(y_{i}-y_{j}\right)}^{2}+{\left(z_{i}-z_{j}\right)}^{2}}\right) \end{array},$$

where *x*, *y*, and *z* represent the Cartesian coordinates of the carbon atoms. Apparently, Equation (4) is not applicable when *i* = *j*. In order to obtain the diagonal elements, the charges are supposed to be distributed uniformly on a triangular surface area surrounding the carbon atom. The area of a triangle is *s* = *3*$\sqrt{3}$*b^2^*, where *b* = 0.142 nm is the length of the C–C bond. The charge density of the triangle area can be expressed as *d* = *q_i_/s*. The electric potential of the atom can be expressed as

(5) $$V{\left(r_{i}\right)}_{i}=\iint_{s}q_{i}/\left(4\pi \varepsilon_{0}s\sqrt{\xi^{2}+\eta^{2}}\right)d\xi d\eta ,$$

The diagonal elements can be obtained:

(6) $$\begin{array}{cc} a_{ii} & =1/\left(4\pi \varepsilon_{0}s\right)\iint_{s}1/\sqrt{\xi^{2}+\eta^{2}}d\xi d\eta \\ & =b\sqrt{3}\text{ln}\left(2+\sqrt{3}\right)/\left(4\pi \varepsilon_{0}s\right) \end{array},$$

The total charges *Q* on a nanorope are

(7) $$Q=\sum_{i=1}^{n}q_{i},$$

The electric potential in the middle of the nanorope is taken as 0. The charge distribution of the nanorope can be obtained by solving Equations (3) and (7) using a MATLAB program.

## 3. Calculation of Adsorption of Metal and Negative Ions

Based on the contact mechanics, the CNTs of the nanorope pile onto each other during the twisting process, and CNTs with larger chiralities produce more marked distortion. CNT nanoropes with small extrusion deformation possess more porosity, which is beneficial for ions entering. Here, the nanorope including CNTs with less chirality (3, 3) was used to simulate the adsorption of metal ions and negative ions.

The molecular dynamics software LAMMPS was adopted to simulate the adsorption and desorption of ions. The NVT ensemble was used for the whole system, and the temperature was 300 K. The L-J pair style potential and Coulomb force were applied to describe the interaction between atoms [28]. The time step was set to be 1 fs and the simulation time was 200 ps. According to our previous study, the large strength of the electric field can cause the nanorope to rotate back to the CNT bundle over a long period of time [28]. In order to reduce the deformation of the nanorope caused by the applied electric field, the electric strength was set to be 1 × 10^−5^ V/Å. After adsorption, we increased the strength of the electric field to realize the desorption. In the present work, we denoted A^+^ with one positive charge to represent Na^+^ or other metal ions as well as B^−^ with one negative charge to describe Cl^−^ or other ions. The nanorope included 6888 carbon atoms, and its combination with 28 A^+^ or 28 B^−^ was simulated. The conditions of the adsorption of the metal and negative ions are listed in Table 1.

The amount of total charge on the nanorope had an effect on ionic adsorption. The adsorption was trivial when there were few charges on the nanorope. As charges increased, a mutation would appear in a narrow scope. During the scope, the adsorption increased from 0% to 100%. In the simulation, a total charge of 4 × 10^−4^ e was chosen. The reason is that it is small to reduce the effect of an initial charge, and the nanorope possessed good adsorption ability with the charge. Trajectory tracking indicated that ions around the charged nanorope were influenced by the applied electrostatic field.

**Figure 2:**
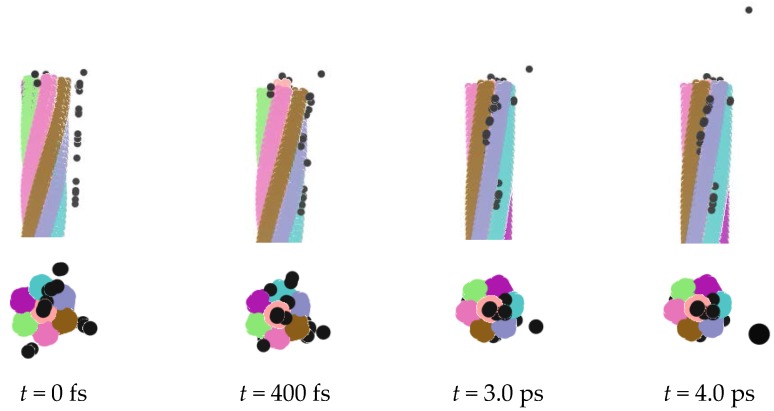
Adsorption of metal ions.

The ionic configuration diagrams are shown in Figure 2 when the simulation time varies from 0 fs, to 400 fs, to 3.0 ps, and to 4.0 ps. The ions in a gray color are adsorbed into nanorope from the top and side directions. The top view shows that, when the ions expanded into nanorope, some may have existed in the gap between the CNTs, and others may have entered into the inside of the CNTs. The side view displays clearly how the ions filled the nanorope along the axial direction. In the initial stage of adsorption, ions were distributed on the surface of the nanorope. After a while, the ions accepted the geometric modulation of the nanorope, and most ions began to enter the rope. Some ions were pushed along the ropes in one direction, close to a CNT. Few ions drifted away due to the long distance from the nanorope.

**Figure 3:**
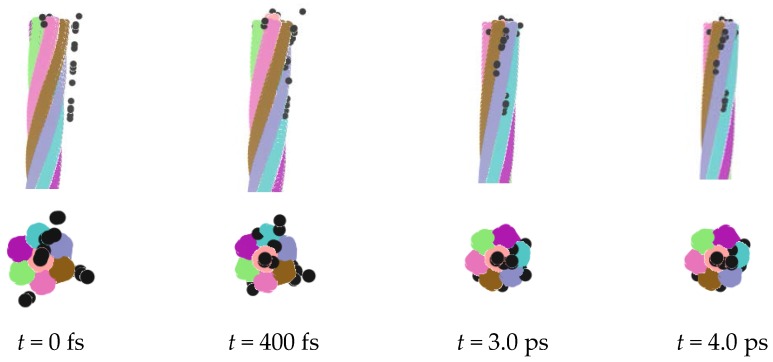
Adsorption of negative ions.

Similarly, the charged nanorope adsorbed negative ions as well. We replaced the metal ions with the negative ones, and all other conditions remained unchanged to perform the simulation. The process of the adsorption of negative ions is shown in Figure 3, which is similar to that of the metal ions. The ions distributing on the surface accepted the geometric modulation and entered the rope in time. The difference is that all negative ions could be adsorbed by the rope.

The adsorption of the negative ions was better than that of metal ions, which may be caused by the charge distribution of the whole system. It can be seen from Figure 2 and Figure 3 that some adsorbed ions may have amassed in the rope under the action of Van der Waals force and static electricity, which can lead to a local increase in ionic density. We also found that the arrangement of adsorbed ions was influenced by the geometrical structure of the nanorope. The control of the ionic adsorption and storage of the nanorope could be realized by changing the strength of the electric field or by applying different charges.

## 4. Adsorption Capacity of Nanorope

**Figure 4:**
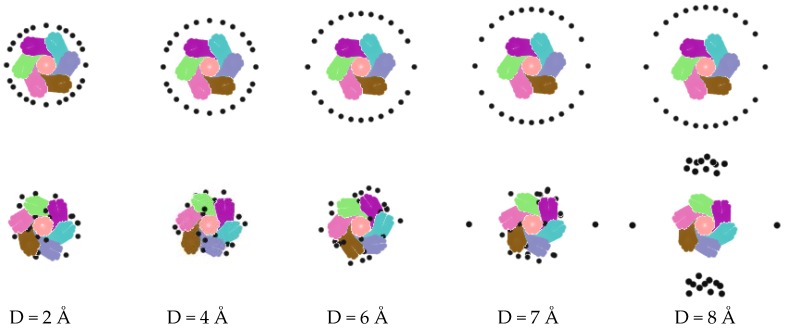
Relation between distance and adsorption.

**Figure 5:**
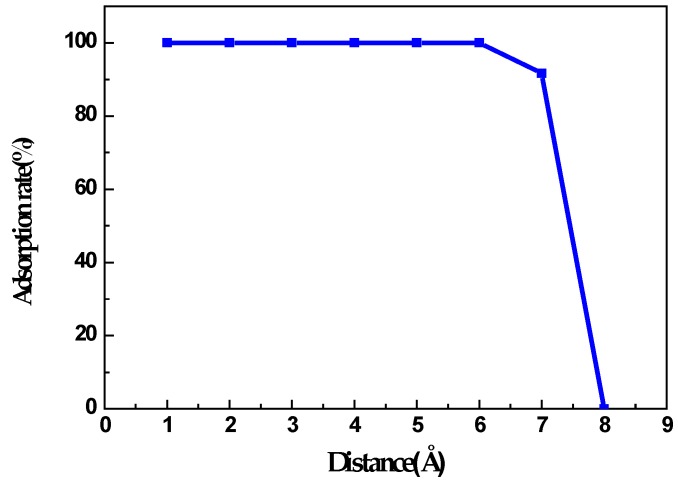
Variation of adsorption rate with distance.

The relative distance between ions and nanorope has an important influence on the adsorption capacity of the nanoropes. The ions distant from the nanorope could not be adsorbed. In this section, we describe how 24 metal ions were used to investigate the influence of the distance on the adsorption capacity of the nanorope. The ions were in random distribution around the rope along the length direction and formed a circle in a cross section. The distance between the ions and the nanorope were 2 Å, 4 Å, 6 Å, 7 Å, and 8 Å, as is depicted in Figure 4. Figure 4 indicates that each ion with a distance less than 6 Å could be adsorbed by the nanorope. However, with the increase in distance, the adsorption of the nanorope was significantly reduced, especially as the distance exceeded 6 Å. The numerical results show that the maximal adsorption rate of 7 Å was 91.7%, and the adsorption rate could plunge when the distance was 8 Å. It can be concluded that, when the distance exceeds a certain value, the charged nanorope can no longer adsorb ions. Figure 5 visually demonstrates the variation of the adsorption rates of the nanorope with the distance between the nanorope and the ions.

**Figure 6:**
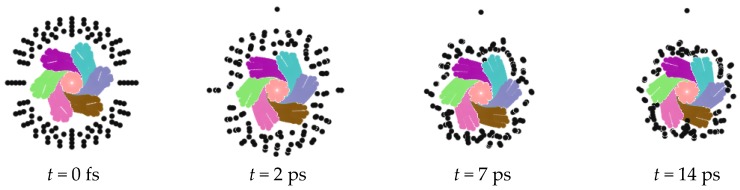
Ionic adsorption capacity of a nanorope.

Although ions can be adsorbed by a nanorope within a certain distance, the amount of ions should be limited. To determine the ionic adsorption capacity of a nanorope, 120 ions were located around a nanorope with a distance less than 6 Å, as shown in Figure 6. Along the length of the CNT nanoropes, the ions were distributed at random. Compared with the example with 24 metal ions, the adsorption rate was no longer 100%, and the process needed more time.

## 5. Desorption of Negative Ions

**Figure 7:**
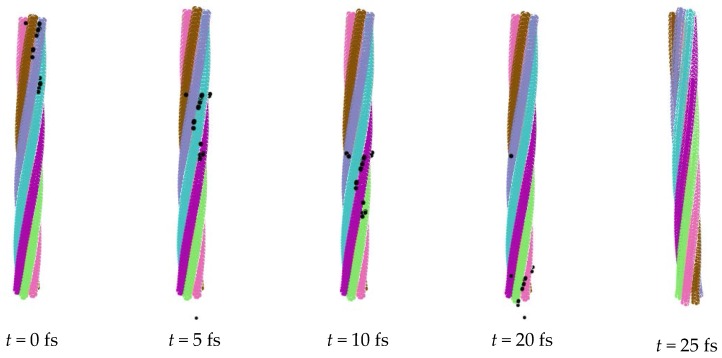
Desorption of negative ions.

Desorption of ions is a reversible process of adsorption, and the ions need to be released from the nanorope. Here, the rope with 28 adsorbed negative ions was used to simulate the desorption process. It was found that, with the increase of the applied electric field, the electric field force was larger than the Van der Waals force and the electrostatic force, which led to the desorption of ions. The strength of the electric field was set to 2 V/Å, and other simulation conditions were the same as the adsorption ones. Figure 7 illustrates the releasing process of the ions. It can be seen that the twisted nanorope rotated back to some extent, and the ions promptly deviated from the rope. We can see that full desorption was shorter when the strength of the electric field was greater. However, because the highest strength of the electric field subjected to the nanorope was never more than 3 V/Å [28], we had to control the strength of the electric field in a certain range to satisfy the desorption demand. After the desorption process, the nanorope was rebuilt by applying a twist load, which demonstrated the recycle of the nanorope.

## 6. Conclusions

In this paper, we built a molecular dynamics model of a nanorope by twisting a CNT bundle. The charge distribution of the nanorope was calculated using a modified gradient method based on classical electrostatic theory. For the charged nanorope, the adsorption and desorption of metal ions and negative ions were achieved by adjusting the strength of the applied electric field. Two factors that influenced the adsorption capacity of the nanorope were found: One is the relative distance between the ions and the rope, and the other is the amount of ions. The results indicate that the nanorope cannot absorb ions distant from it. As the amount of ions increase, adsorption reaches its limit, and process time is prolonged. The strength of the electric field can be taken as an adjusting switch, making it possible to provide a standard system for the adsorption and desorption of ions at the nano scale. This is very significant for applications in sewage treatment.

## Figures and Tables

**Table 1:** The conditions of adsorption of metal and negative ions.

Conditions	A^+^	B^−^	C-atom	Ensemble	Time
1	28	0	6888	NVT	200 ps
2	0	28	6888	NVT	200 ps
